# The *FTO* gene polymorphism rs9939609 is associated with obesity and disability in multiple sclerosis patients

**DOI:** 10.1038/s41598-019-55742-2

**Published:** 2019-12-13

**Authors:** Ahmad Al-Serri, Raed Alroughani, Rabeah A. Al-Temaimi

**Affiliations:** 1 Human Genetics Unit, Department of Pathology, Faculty of Medicine, Kuwait University, Kuwait, PO Box 24923, Safat 13110 Kuwait; 2 Division of Neurology, Department of Medicine, Amiri Hospital, Kuwait City, Kuwait

**Keywords:** Genetic association study, Multiple sclerosis, Obesity

## Abstract

Obesity is a well-known risk factor for multiple diseases including multiple sclerosis (MS). Polymorphisms in the *fat-mass obesity* (*FTO*) gene have been consistently found to be associated with obesity, and recently found to increase the risk of developing MS. We therefore assessed the common *FTO* gene polymorphism (rs9939609) in relation to obesity, risk of developing MS and its disability in a cohort of MS patients. A cohort of 200 MS patients (135 females and 65 males) were genotyped for the *FTO* rs9939609 polymorphism. Using both logistic and linear regression we assessed the relationship between the variant and the selected phenotypes under both an additive and recessive genetic models. The A-allele was found to be associated with being overweight/obese in MS patients (OR = 2.48 (95% CI 1.17–5.29); p = 0.01). In addition, The A-allele was also found to be associated with increased MS disability (β = 0.48 (95% CI 0.03–0.92); p = 0.03). However, no association was found with risk of developing MS (p > 0.05). Moreover, our association with obesity is consistent with previous reports, whereas the association with disability is novel and warrants further investigation on the role of *FTO* in disease progression.

## Introduction

Multiple sclerosis (MS) is a chronic demyelinating autoimmune disorder of the central nervous system with over 2.2 million people affected worldwide^1^. The average MS age of onset is between 20–40 years of age, however MS has been reported to occur in younger and older ages as well^2,3^. There is no specific etiology for the development of MS, however both genetic and environmental factors have been found to contribute to its development^4,5^. Obesity is an environmental risk factor for multiple diseases including MS^6–8^. These studies have been conducted on both children and adults and have shown a 2-fold increase in the development of MS in obese (Body mass index (BMI) > 30 kg/m^2^) subjects compared to subjects with BMI between 18.5–21 kg/m^2^ ^9^. In addition, obesity has been shown to associate with worsening MS disability, and a faster conversion of relapsing-remitting MS (RRMS) to secondary progressive MS (SPMS)^10–12^. With studies implicating the low-grade neuroinflammation associated with obesity to contribute to MS disability progression^13,14^. MS disability monitoring measures the change in disability over time in MS patients that is an important guide for clinicians to stratify patients progress, make disease management decisions and early therapeutic interventions. The expanded disability status scale (EDSS) has been used extensively since its development to guide in diagnosing MS disease clinical course and designing and monitoring treatment regimens to slow down MS disease progression^15^.

The prevalence of obesity has tripled in the past 30 years and it is estimated to be 50% in the Kuwaiti population^16^. Both genetic and environmental factors contribute to the development of obesity^17^. In 2007 a genome-wide association study (GWAS) found a single nucleotide polymorphism (SNP) in the *fat mass obesity* (*FTO*) gene to be associated with increased BMI in both children and adults^18,19^. The variant (rs9939609) today has been consistently associated with obesity in multiple populations making *FTO* the most commonly studied obesity gene^18,20^. The FTO is an alpha-ketoglutarate dependent dioxygenase that is highly expressed in the nucleus. While the exact function of this protein is unknown it is reported to be an RNA demethylase that mediates demethylation of different RNA species including mRNAs, tRNAs and snRNAs^21,22^. However, the most studied role of FTO is as a regulator of fat mass, adipogenesis and energy homeostasis^23^. FTO is believed to be involved in multiple pathways including; DNA/RNA repair^24^, adipogenesis^25^, thermogenesis^26^, and neurogenesis^27^. *FTO* genetic variants and altered expression have been reported to associate with several obesity-related chronic diseases such as type-2-diabetes^28^ and cancer^29^. In addition, *FTO* genetic variants have been found to affect disease outcome and risk in obese patients with other diseases such as psoriasis^30^, polycystic ovaries syndrome^31^, and cardiovascular diseases^32^. Interestingly, only two studies to date have investigated *FTO* variants in MS patients^9,33^.

Based on the association of obesity with MS risk and the established association of *FTO* rs9939609 with obesity, investigating obesity genes in relation to MS should be conducted to understand the mechanism underlying the link between the two diseases. Therefore, in this study we were interested in assessing the relationship between the common *FTO* gene polymorphism (rs9939609) with obesity, MS risk and disability in a cohort of MS patients.

## Results

### Assessing rs9939609 in relation to MS risk and BMI status in MS patients

The MS population sample in this study included 135 females and 65 males (n = 200), whereas healthy controls included 125 females and 91 males (n = 206). The demographics and clinical characteristics are shown in Table 1.

**Table 1:** MS cohort’s demographics and clinical characteristics.

Criteria	MS cohort (n = 200)	Controls (n = 206)	p-value
**Sex (%)**			
Female	135 (67.5)	125 (60.5)	0.152
Male	65 (32.5)	81 (39.5)	
Age in years (Average [±SD^a^])	32.32 ± 9.35	52.39 ± 8.28	<0.0001
BMI^b^ (Average [±SD])	27.32 ± 6.29	31.52 ± 7.14	<0.0001
**BMI categories (%)**			
Underweight	9 (4.5)	0 (0)	<0.0001
Normal weight	73 (36.5)	24 (11.5)	
Overweight/Obese	118 (59)	182 (89.5)	
MS type (%)		NA	
Benign MS	9 (4.5)	—	
RRMS^c^	171 (85.5)	—	
SPMS^d^	17 (8.5)	—	
PPMS^e^	3 (1.5)	—	
EDSS^f^ (Average [±SD])	2.377 ± 1.75	NA	
Disease duration in years (Average [±SD])	5.69 ± 5.22	NA	

^a^SD: Standard deviation; ^b^BMI: Body mass index; ^c^RRMS: Relapsing-remitting MS; ^d^SPMS: Secondary progressive MS; ^e^PPMS: Primary progressive MS; ^f^EDSS: Expanded disability status scale; NA: Not applicable.

*FTO* rs9939609 genotype distribution in the MS cohort did not differ from the sampled healthy Kuwaiti population distributions after adjusting for both age and gender under both an additive and recessive genetic model (p = 0.33, 0.23 respectively) (Table 2). In addition, healthy controls and MS cohort rs9939609 genotype distribution were in Hardy-Weinberg Equilibrium (p = 0.44 and 0.42, respectively).

**Table 2:** Assessing the risk of developing MS by comparing the *FTO* polymorphism between healthy controls and MS patients under both an additive and a recessive genetic model.

SNP	Model	Controls	MS	OR^a^ (95% CI^b^)	p-value
*FTO* rs9939609	Additive				
	TT	62 (30.1%)	59 (29.5%)		
	TA	97 (47.1%)	94 (47%)	1.25 (0.8–1.94)	0.33
	AA	47 (22.8%)	47 (23.5%)		
	Recessive				
	TT/TA	159 (77.2%)	153 (76.5%)		
	AA	47 (22.8%)	47 (23.5%)	1.57 (0.74–3.32)	0.23

^a^OR: Odds ratio; ^b^CI: Confidence interval.

We assessed the relationship between the common *FTO* gene polymorphism and obesity status in MS patients after adjusting for both age and gender (Table 3). In a dose-dependent trend the A-allele was found to be associated with overweight/obesity (p = 0.04). Moreover, using a recessive genetic model a significant difference (p = 0.01) was observed in the distribution of the AA genotype in the overweight/obese group compared to the BMI normal group, 29.9% compared to 14.5% respectively (Table 3).

**Table 3:** Relationship between the *FTO* polymorphism and obesity status in MS patients using both an additive and a recessive genetic model.

SNP	Model	Normal weight	Overweight/obese	OR^a^ (95% CI^b^)	p-value
*FTO* rs9939609	Additive				
	TT	28 (33.7%)	31 (26.5%)		
	TA	43 (51.8%)	51 (43.6%)	1.51 (1–2.28)	0.04
	AA	12 (14.5%)	35 (29.9%)		
	Recessive				
	TT/TA	71 (85.5%)	82 (70.1%)		
	AA	12 (14.5%)	35 (29.9%)	2.48 (1.17–5.29)	0.01

^a^OR: Odds ratio; ^b^CI: Confidence interval.

### Assessing the relationship between rs9939609 and EDSS

The relationship between the common *FTO* gene polymorphism and EDSS showed a significant dose-dependent association (p = 0.04) when adjusted for gender and disease duration (Table 4). A recessive genetic model showed carriers of the AA genotype had 0.67 higher EDSS compared to those with both TT and TA genotypes combined (p = 0.03) (Table 4). The association remained significant after adjusting for gender, disease duration, age and BMI categories (p = 0.037 for the additive model and p = 0.025 for the recessive model).

**Table 4:** Relationship between the FTO polymorphism and EDSS using both additive and recessive genetic models.

SNP	Model	EDSS^a^	β^b^ (95% CI^c^)	p-value
*FTO* rs9939609	Additive			
	TT	2.09 ± 0.21		
	TA	2.18 ± 0.16	0.26 (0.009–0.52)	0.04
	AA	2.72 ± 0.31		
	Recessive			
	TT/TA	2.15 ± 0.12		
	AA	2.72 ± 0.31	0.48 (0.03–0.92)	0.03

^a^EDSS: expanded disability status scale; ^b^β: beta-coefficient; ^c^CI: confidence interval.

## Discussion

In this study we report an association between *FTO* rs9939609 gene polymorphism and risk of obesity in a cohort of MS patients. In addition, we found the *FTO* risk allele to associate with increase in disability of MS patients. Although we did not find an association between the *FTO* variant and risk of developing MS when compared to controls, our finding is consistent with the previous study showing no association between *FTO* rs9939609 in MS patients^33^. The study by Davis *et al*., reported the risk allele of rs9939609 not to be associated with MS risk however is associated with increased homocysteine levels in MS patients compared to controls^33^. However, a recent study by Gianfrancesco *et al*., found a direct effect between another *FTO* variant (rs1558902) and risk of developing MS in Hispanics^9^. Therefore, our inconsistency and lack of association between the *FTO* rs9939609 and MS risk can be due to the differences in the variant selected and the population studied. Being overweight and obese is endemic to the Kuwaiti population, specifically in Kuwaiti nationals; which potentiates a significant health risk and predisposition to obesity-related diseases in the population^34^. Several studies have repeatedly shown that obesity is a risk factor for early-onset MS^35–37^. While the underlying mechanism by which obesity contributes to MS etiology remains unclear several hypotheses have been postulated including; the effect of persistent low-grade neuroinflammation^38^, the role of adipose tissue adipokines in promoting inflammation^39^, and obesity-related central nervous system cardiovascular alterations^40^. The role of obesity in MS expands beyond risk as it has been shown to worsen MS disease disability and progression as well^10,13^.

*FTO* rs9939609 has consistently been shown to associate with higher BMI across different populations including the Kuwaiti population^18,20^. The effects of rs9939609 has been rigorously investigated since its discovery for its functional impact in relation to obesity. Reported evidence points to a role in food craving behavior since FTO is highly expressed in the hypothalamus that controls the regulation of food intake^41,42^. Another evidence suggests rs9939609 has direct influence on muscle mass and oxidative potential as it was found associated with decreased slow-twitch muscle fibers in lean sport athletes and lower muscle lean mass in non-resistance trained athletes^43,44^. In addition, it has been shown that MS affects skeletal muscles resulting in fewer and smaller muscle fibers and lower lean muscle mass^44^. Collectively, rs9939609 functional roles may explain the association of the A allele with MS disability. It is plausible that MS carriers of the A allele have less cognitive restraint towards food intake and physical activity commitment. Lower cognitive restraint may impact an already poor lean muscle mass in these patients reducing their inclination to perform physical exercise. This possibility is amplified by the psychological impact of their MS diagnosis which may ultimately result in increases in body fat and less physical activity that has been shown to directly impact disability in MS patients^45,46^. Moreover, the average EDSS score increase for all RRMS patients with EDSS scores between 0–6 has been reported to be 0.168 per year suggesting that A allele carriers might not be recognized at risk of rapid disability development at the lower EDSS points (0–3) but are at a significant risk at higher ends of the scale (>3.5)^47^. The clinical implication of A allele carriers having an 0.67 increase in EDSS after adjusting for disease duration should guide the clinical design of treatment regimens for this specific subset of MS patients to counter its influence early on MS progression as timing is of importance in MS disease clinical management^48^.

Interestingly, recent evidence of FTO’s role in adult neurogenesis and the association of an *FTO* variant (rs1558902) with MS risk points to a possible direct neuronal function for FTO in MS relapse-repair and remyelination^9,27^. Moreover, the study by Smemo *et al*., demonstrates a connection between *FTO* variants and the homeobox *IRX3* gene which is a transcription factor that is highly expressed in the brain^49^. The *IRX3* gene is involved in body mass and composition, and is suggested to be regulated by *FTO* variants^49^.

Although clinical data was well defined and available for this MS cohort however the current study has its limitations. The modest sample size suggests the importance of replication studies on a larger and independent cohort before a putative conclusion on the role of FTO in MS progression can be drawn.

In conclusion, this is the first study as far as we are aware to report MS patients carrying *FTO’s* risk allele are at a greater risk of MS disability progression and therefore should be encouraged to adapt to higher cognitive restrains towards food craving and higher commitment towards physical activity for better disease outcomes.

## Methods

### Patients sample selection and ethics

Two hundred Kuwaiti MS patients were recruited for this study at Dasman diabetes institute MS clinic. The study protocols were approved by Dasman diabetes institute ethical review committee and Health Sciences Center ethical committee, Kuwait, both of which adhere to the declaration of Helsinki Ethical Principles for Medical Research Involving Human Subjects. All study protocols and objectives were fully explained to all participants before securing informed written consent. MS patients’ inclusion criteria were as follows; having a confirmed MS diagnosis according to the standardized modified McDonald’s criteria^50^, a detailed clinical history (demographics, BMI measurement, age of MS onset, disease duration, expanded disability status scale (EDSS) score, and treatment history), being a Kuwaiti citizen, and an MS disease duration of ≥2 years. All magnetic resonance imaging (MRI) and EDSS assessments were conducted by an experienced neurologist in accordance with the standard approved EDSS method^51^. A 4 mL blood sample was collected from each patient in an EDTA coated vacutainer. Collected blood samples were centrifuged at 2,500 × g at room temperature for 10 minutes and buffy coat fractions were collected and stored at −20 °C until use.

### FTO rs9939609 genotyping

Qiagen DNA mini kit (Qiagen, CA, USA) was used for buffy coat DNA extraction with minor modifications. In summary, 200 stored frozen buffy coat samples from MS patients were thawed in a water bath set at 37 °C, and 200 µl aliquots were transferred to a sterile microcentrifuge tube. Samples were digested at 56 °C with proteinase K and lysis buffer for 20 minutes. Absolute ethanol was added to stop sample digestion and precipitate proteins. Samples were eluted through a QIAamp mini spin filter columns to bind isolated DNA to the filter membranes. Columns were washed twice with wash buffers of differing stringency, and dried by spinning at 14,000 × g for one minute followed by air drying for 2 minutes. DNA was eluted with 150 µl of Tris-EDTA buffer. All centrifugations were performed at room temperature. DNA yield and quality were assessed using a NanoDrop spectrophotometer. *FTO* SNP rs9939609 was assessed using Taqman rs9939609 genotyping assay according to standard manufacturer protocol (Life Technologies, CA, USA). In brief, 50 ng of DNA was used in a total of 20 µl reaction mixture inclusive of 1x TaqMan Universal PCR Master Mix and 1x probes/primers mix. Reactions were loaded in a 96-well plate and a plate pre-PCR read was recorded in an ABI 7500 Fast Real-time PCR system (Life technologies, CA, USA). PCR was performed under the following conditions; a hold step at 95 °C for 10, and 45 cycles of denaturation at 92 °C for 15 seconds followed by annealing/extension at 60 °C for 1 minute. Post-PCR read was recorded for end-point fluorescence detection. Allelic discrimination analysis was performed and analyzed using ABI 7500 Fast Real-time PCR system SDS software (Life technologies, CA, USA).

### Statistical analysis

*FTO* rs9939609 Kuwaiti population frequencies from a previous study were used to compare to MS cohort allelic and genotype distributions^20^. A subset of healthy Kuwaitis was selected from that study’s population according to the following criteria; no history of any chronic disease (i.e. diabetes), being ≥40 years old to exclude individuals that may have a future MS diagnosis, and a comparable sex ratio to our MS cohort. MS patients characteristics were expressed as mean ± standard deviation (SD) and percentages where appropriate. Hardy-Weinberg equilibrium (HWE) was calculated. Association between the variant and MS risk, obesity status was assessed using binary logistic regression whereas linear regression was used to assess the association with EDSS expressed as odds ratio (OR) and Beta-coefficient (β) respectively. Both an additive and recessive genetic models were used to assess the dose-dependent association and the impact of having two risk-alleles. All statistical analyses were performed using SPSS software (version 25; SPSS Inc, Chicago, IL, USA) and “SNPassoc” package from R where appropriate^52^. Significance was set at p < 0.05.

## Data Availability

The raw genotypic data is available for any meta-analysis studies upon request.
